# The Development and Evaluation of a Vocational Rehabilitation Training Programme for Rheumatology Occupational Therapists†

**DOI:** 10.1002/msc.1050

**Published:** 2013-05-24

**Authors:** Rachel O'Brien, Sarah Woodbridge, Alison Hammond, Julie Adkin, June Culley

**Affiliations:** 1 Sheffield Hallam UniversitySheffield, UK; 2 Royal Derby HospitalDerby, UK; 3 School of Health Care Sciences, University of SalfordSalford, UK; 4 Department for Work and PensionsDerbyshire; 5 Derby Branch of the National Rheumatoid Arthritis Society

**Keywords:** Vocational rehabilitation/ work long term conditions rehabilitation

## Abstract

People with inflammatory arthritis rapidly develop work disability, yet there is limited provision of vocational rehabilitation (VR) in rheumatology departments. As part of a randomized, controlled trial, ten occupational therapists (OTs) were surveyed to identify their current VR provision and training needs. As a result, a VR training course for OTs was developed which included both taught and self-directed learning. The course included: employment and health and safety legislation, work assessment and practical application of ergonomic principles at work.

Pre-, immediately post- and two months post-training, the ten OTs completed a questionnaire about their VR knowledge and confidence On completion, they reported a significant increase (p < 0.01)in their knowledge and confidence when delivering vocational rehabilitation. They rated the course as very or extremely relevant, although seven recommended more practical sessions. The preference for practical sessions was highlighted, in that the aspects they felt most beneficial were role-playing assessments and sharing ideas through discussion and presentations.

In conclusion, the course was considered effective in increasing both knowledge and confidence in using VR as an intervention, but, due to time constraints within the working day, some of the self-directed learning should be incorporated into the training days. Copyright © 2013 John Wiley & Sons, Ltd.

## Introduction

Work or vocational rehabilitation (VR) is defined as ‘a process to overcome the barriers an individual faces when accessing, remaining or returning to work following injury, illness or impairment’ [Department of Work and Pensions (DWP), 2004]. Inflammatory arthritis [such as rheumatoid arthritis (RA), psoriatic arthritis (PA) and undifferentiated early inflammatory arthritis (EIA)] commonly causes work problems and work disability. For example, within five years of diagnosis, 28–40% of people with RA stop work (Eberhardt et al., 2007). Before becoming work disabled, people with inflammatory arthritis experience work instability (i.e. a mismatch between their abilities and job demands which threatens employment). Pain, fatigue and limited hand function are particular problems (Allaire et al., 1996, 2006). Reduced productivity (i.e. reduced work performance) and increased sick leave are common (Zhang et al., 2008; Zirkzee et al., 2008). Once out of work, people with inflammatory arthritis are much less likely to return to work (Verstappen et al., 2004; Young et al., 2009). It is therefore better to identify work problems early and help people stay at work by providing job retention VR.

VR has been part of occupational therapy (OT) since the early days of the profession. The College of Occupational Therapists’ VR Strategy (2008) states that OTs should routinely ask service users about work, and offer VR services. Important skills are conducting work interviews, task analysis, recommending job accommodations (i.e. modifications and adjustments), providing work rehabilitation and work hardening. OTs in the UK have most of the necessary skills but often little or no VR experience. OT secondary care services in particularly have become focused on improving independence in the activities of daily living. There is little time for OTs to address the increasing social participation of clients through providing VR, although it is an important part of the management of people with long-term conditions.

Politically, there is increasing recognition of the contribution that VR can make in preventing people with long-term conditions from becoming unemployed, claiming social security benefits and having poorer health outcomes due to loss of role and decreased income (Black, 2008; Black and Frost, 2011; Boorman, 2009). Rheumatology OTs are well placed to identify people with inflammatory arthritis who have work problems. Their role includes evaluating the impact of arthritis on functional ability, work and social roles. Thus, people working, but experiencing work difficulties, could be identified early. OTs could provide job retention VR to help people modify their work to suit their abilities, whether or not they choose to disclose their condition to their employer. However, although rheumatology OTs can provide job retention VR, the availability and quality in the UK varies considerably. It ranges from a brief intervention providing information booklets, ‘signposting’ about services available and general ergonomic advice, to a thorough work assessment and VR intervention including:

- Job accommodations, such as ergonomic workstation interventions, job redesign, assistive technology, adaptations and working splints;
- Work-related joint protection and fatigue management training;
- Psychosocial and informational strategies (e.g. increasing confidence to ask for job accommodations at work, disclosing about arthritis to employer and work colleagues, employment rights, statutory VR service availability);
- Coping strategies, such as stress management and relaxation training;
- Work site visits;
- Liaising with employers about: flexible working hours, education about arthritis; and availability of occupational health and statutory VR services (such as Access to Work and Disability Employment Services) to support workers;
- Multidisciplinary team referral to manage the condition.

There is some evidence such rheumatology OT job retention VR services for people with inflammatory arthritis are effective. A randomized, controlled trial (RCT) with working people with RA (*n* = 32) in the UK identified that, at six months, the VR group had significantly improved self-perceived ability to manage self-care, work and leisure, work instability, work satisfaction and pain compared with a waiting list control group (Macedo et al., 2009). An RCT in the USA evaluated the VR provided by VR counsellors, which was similar to that which OTs might provide (Allaire et al., 2003). People with arthritis (RA, PA, knee osteoarthritis, systemic lupus erythematosus or ankylosing spondylitis) (*n* = 242) received either information booklets about managing health-related employment problems and work services or the booklets plus two 1.5-hour individual VR meetings. A structured interview [the Work Experience Survey – Rheumatic Conditions (WES-RC)] was used to explore job barriers and develop an action plan of job accommodations to overcome these (Allaire and Keysor, 2009). Information on employment rights, disclosing arthritis to employers and asking for job accommodations was also given. At 3.5 years, there were significantly fewer job losses in the VR group.

As part of a pilot RCT evaluating the effectiveness of ajob retention VR intervention for people with inflammatory arthritis (funded by Arthritis Research UK), we aimed to devise and evaluate a short training programme for rheumatology OTs, to enhance their ability to provide job retention VR. The study included:

Phase 1: Conducting a survey with rheumatology OTs about their current job retention VR service;
Phase 2: Developing a job retention VR training programme and resources to enhance the assessment and VR intervention skills of rheumatology OTs;
Phase 3: Evaluating the effect of this training on the OTs’ self-perceived knowledge and self-efficacy to deliver job retention VR.

## Phase 1: Survey of OTs’ current VR provision

This was conducted to investigate rheumatology OTs’ current VR experience and training needs, and inform the VR training programme development process.

### Method

Ten OTs, due to deliver the trial VR intervention, were surveyed about their VR provision. Closed questions included: NHS band; length of rheumatology experience; how many patients with inflammatory arthritis they treated each month; how many of these experienced work problems; duration of VR provided; and how often they conducted work site visits. Open questions asked about the nature of the VR provided.

### Results

All ten OTs completed the questionnaire. Six were Band 6, two Band 7 and two Band 8. They had 8.5 [standard deviation (SD) 4.10] years of rheumatology experience and provided VR for four [interquartile range (IQR) 1–9] years. On average, they each treated 21.30 (SD 11.82) patients with inflammatory arthritis per month, of whom a quarter (5.09, SD 4.01) had work problems, most of whom (3.90, SD 1.29) were still employed. None received VR referrals specifically from rheumatology clinics. Patients’ work problems were identified during OT assessment.

For employed clients, the OTs provided 45 (IQR 30–90) minutes of VR over 1.5 (IQR 1–2) treatment sessions. None conducted a standardized work assessment, although four reported discussing task analysis with clients to identify work problems. All provided patients with written information: six were given the National Rheumatoid Arthritis Society (NRAS) ‘I want to work’ (2011) booklet; and four the Arthritis Research UK ‘Work and arthritis’ (2010) booklet. Two additionally provided written information about workstation adaptation and local VR services. The VR provided is summarized in Table 1. Occasionally, three OTs had conducted work site visits with clients and two liaised with employers, providing information about clients’ work-related problems and solutions.

**Table 1:** Occupational therapists’ vocational rehabilitation provision (*n* = 10)

VR component (n)	Comments (n)
Ergonomic (joint protection) advice (*n* = 10)	Altering workstations (*n* = 6)
	Adaptive equipment (*n* = 5)
	Posture/positioning (*n* = 4)
	Discussing basic ergonomic principles (*n* = 1)
Fatigue management (*n* = 9)	Pacing (e.g. taking short breaks) (*n* = 7)
	Rotating tasks regularly (*n* = 4)
	Changing work patterns and shift duties (*n* = 3)
‘Signposting’ to other services (*n* = 8)	Self-referral to Access to Work scheme and/or Disability Employment Advisory Service (*n* = 3)
	Occupational health services at work (*n* = 3)
Practical activities (*n* = 4)	Applying principles to a computer workstation analysis (*n* = 2)
	Trying out adaptive equipment (*n* = 4)
	Psychological interventions (stress management and relaxation) (*n* = 2)
Disclosure (*n* = 1)	How to discuss arthritis with employers (*n* = 1)

## Discussion

Rheumatology clinic staff at the participating sites were not referring clients with work problems for VR. Consequently, OT services were not configured to meet this need. The OTs were unfamiliar with standardized work assessments but some did briefly analyse clients’ jobs. Although all provided brief work-related ergonomic and fatigue management advice, few included practical activities. Written advice and signposting about other services were common but work site visits, employer liaison, employment rights advice and discussions about disclosing arthritis to employers were not. OTs commented that there was little time to provide VR and to develop VR skills. These results helped to inform the content of the training programme.

## Phase 2: Development of the job retention VR training programme

During the trial development phase, the research team decided to provide a VR intervention based on that described in Allaire et al. (2003) and Macedo et al. (2009). The trial VR intervention and training programme development group consisted of five OTs, all with VR and teaching experience, a disability employment advisor and a member of the NRAS, who was a working person with RA. The group reviewed the interventions in these studies and identified components to include in the intervention and the training programme. Additionally, the group reviewed VR guidelines (Waddell et al., 2008; College of Occupational Therapists, 2008), the survey results and their own experience as providers and receivers of work rehabilitation for arthritis. From this, areas for training were identified, including:

- Assessment of clients’ physical and psychosocial functioning related to work, work environment, work skills required in their job, and barriers to work;
- Conducting the Work Environment Survey – Rheumatic Conditions (Allaire and Keysor, 2009) to enable this assessment process
- Analysing jobs; for example, task analysis; working positions such as grips, postures, activity cycles [facilitated by using the Ergonomic Assessment Tool for Arthritis (EATA; Backman et al., 2008)]
- Providing solutions to work-based problems; for example, ergonomic modifications to workstations; job redesign; task rotation; modifying manual handling techniques; using different or modified tools, specialist equipment and/or assistive technology; environmental adjustments, transport modifications;
- Applying condition management skills; for example, fatigue management, joint protection, correct posture and positioning, use of splints, stress management) to work activities and enabling people to make behavioural changes at work;
- Negotiation and communication skills to enable clients to achieve key objectives; for example, disclosure of arthritis to the employer, obtaining changes in working hours and duties;
- Current work-related legislation: the Equality Act (2010), Health and Safety At Work Act (1974) and The Management of Health and Safety At Work Regulations (1999);
- Statutory and third-sector employment support and advisory services in the UK; for example, disability employment advisors (DEA), Access to Work (AtW); work-related social security benefits available; and local support groups, such as NRAS;
- How health is managed in the workplace; for example, the role of human resources and occupational health and employer liaison.

Assessing clients’ physical and psychosocial status, environment and interpersonal skills; providing condition management; and facilitating clients in making changes are core OT skills. However, they are more usually applied in activities of daily living than in work. Training was therefore also focused on how existing skills are applied in the work setting. As many of the OTs reported only discussing work problems, the training including practical skills.

The group initially planned a two-day training programme, as the OTs reported that study leave approval for longer periods was difficult to obtain. Topics were prioritized, with greater time allowed for developing WES-RC assessment and solution-generating skills, and practical experience. The topics included are shown in Table 2. A VR resource manual was developed to support OTs in assessing specific work problems (e.g. seating, workstation, manual handling) and problem solving, and as a solutions resource. It also included work and arthritis research articles and summaries of legislation and employment services. OT feedback and programme review determined that further training was necessary. Accordingly, self-study activities were designed to consolidate learning from the programme and help applying this into practice. A third study day was included three months later to address providing feedback on homework; addressing unmet needs; and consolidating learning.

**Table 2:** Relevance of training programme content (*n* = 10)

Topic	Median (IQR)
**Day 1:**	
Introduction to Work and IA trial	5 (4.5–5.0)
Legislation	4.5 (3.75–5.00)
Role of the disability employment advisor	4.5 (4–5)
RA – Service user's perspective	5 (4–5)
Setting the scene in which we work	4 (3.75–5.00)
VR strategies: Upper limb	5 (4–5)
VR strategies – Practical hand tools	4.5 (3.75–5.00)
(Five options: mice, screwdrivers, keyboards, cutting tools, saws)	
VR strategies: Environment	4 (4–5)
VR strategies: Practical – Load handling (five options: roller cage, shovels, trolleys, lifting above shoulder, lifting floor to waist)	5 (4–5)
VR strategies: Disclosure to employers/work colleagues	4.5 ( 4–5)
**Day 2:**	
Completing WES-RC case study (assessment and solutions)	5 (4.75–5.00)
**Study tasks:**	
Doing the EATA	3.50 (3.00–4.25)
Doing the telephone WES-RC	5 (4–5)
Completing WES-RC solutions	5 (4–5)
Preparing VR strategy presentations and hand-out	4 (4–5)
**Day 3:**	4 (4–5)
Discussion of telephone WES-RC case studies	
VR strategy presentations from other occupational therapists	5 (4–5)
Practical: Seating evaluation	5 (5–5)
Practical: Bakery work analysis	4 (4–5)

Key: 1 = not relevant; 2 = a little relevant; 3 = moderately relevant; 4 = very relevant; 5 = extremely relevant

EATA, Ergonomic Assessment Tool for Arthritis; IQR, interquartile range; RA, rheumatoid arthritis; VR, vocational rehabilitation; WES-RC, Work Experience Survey – Rheumatic Conditions.

Different learning and teaching approaches were incorporated into the programme to facilitate different learning styles. These included: short presentations; discussions; working in pairs on case studies completing the WES-RC, developing solution plans and then discussing treatment plans among the larger group; small group work doing practical tasks assessing the ergonomic requirements of tasks and, for example, evaluating tools, adaptive equipment and manual handling required for lifting and moving roller cages; and therapists’ presentations of practical methods of applying existing core skills to the work place (e.g. teaching pacing, joint protection and stress management to enable behavioural change). The homework study activities included: planning to conduct a WES-RC, conducting a telephone WES-RC with one of the course facilitators (SW, RO'B) role-playing a client, completing an ergonomic assessment based on a case study from the training programme (using the EATA), researching tools and equipment available to resolve problems identified from these and familiarizing themselves with the VR resources manual.

## Phase 3: Evaluation of the OT job retention VR training programme

Therapists’ perceptions of the impact the training programme were sought to evaluate its effectiveness and enable further development of the training programme.

### Method

A questionnaire was developed to assess the training programme's impact on OTs’ perceived ability and confidence in delivering VR pre- and post-training. The questionnaire included information on: clinical band and length of rheumatology experience; their knowledge of and confidence in delivering VR components [using a scale of poor (1) to very good (5)]; open-ended questions about the most- and least-liked aspects of the programme; the relevance of each topic covered [rated on a scale of not at all relevant (1) to very relevant (5)]; and suggestions for improvements to the programme. The same questionnaire was completed by participating OTs at the beginning of day 1 and at the end of day 3.

### Results

All ten OTs attended the VR job retention training programme. There was a significant increase (*p* < 0.01) in both self-reported knowledge of and confidence in delivering VR among course participants (Table 3). The OTs rated the training course content as very or extremely relevant (Table 2). The three things considered most beneficial were: sharing ideas/observing other OTs during the OT presentations on day 3 (*n* = 5); role play and completing the telephone WES-RC (*n* = 5); and the practical sessions (*n* = 5). Six considered all of the programme useful. Four commented that they wanted more time, especially in the practical sessions. Seven recommended more practical sessions, with suggestions including: more on manual/production line jobs (*n* = 3); computer workstation equipment (*n* = 1); analysing equipment (*n* = 1); visiting a production line (*n* = 1); having real patients present with whom to complete the case studies (*n* = 1); report writing to managers (*n* = 1); advice on self-employment (*n* = 1).

**Table 3:** Median (IQR) ratings for vocational rehabilitation (VR) knowledge and confidence in providing VR interventions pre- and three months post-VR programme (*n* = 10)

	Pre-VR course	Post-VR course	W	*p*
Knowledge of VR	2	4	–2.86	0.004
	(1.75–3.00)	(3–4)		
Knowledge of VR process	2	4	–2.85	0.004
	(1–3)	(3–4)		
Knowledge of VR strategies	2	4	–2.72	0.006
	(2–3)	(3–4)		
Knowledge of relevant legislation and policy	2	3	–2.76	0.006
	(1.75–3.00)	(2.75–4)		
Confidence in completing a work assessment	2	3.5	–2.55	0.01
	(1–3)	(3–4)		
Confidence in identifying work solutions and strategies	2	4	–2.56	0.01
	(1.75–3)	(3–4)		

**Key:** 1 = poor; 2 = limited; 3 = average; 4 = good; 5 = very good

IQR, interquartile range; W, Wilcoxon signed rank test.

Nine commented about the self-directed study: that it took longer than the planned equivalent of a day (at about ten hours); that it had been difficult to fit into their working days in the three months between the training days; and that there was too much self-directed learning. One recommended that the self-directed study should be included within a training day. Of the five commenting about programme duration: three thought four or five days would have been better, but two that three days was about right.

## Discussion

The aim of our research was to develop and evaluate a job retention VR training programme for OTs working with people with inflammatory arthritis. Ten OTs specializing in rheumatology completed the programme. Prior to the training, the OTs felt that they lacked knowledge of and confidence in delivering VR. Historically, VR has been a key OT intervention, encompassing the core OT skill of activity analysis. However, during the last 15 years the focus on rehabilitation to improve participation (including employment) has reduced in many areas of OT, with emphasis placed on short episodes of treatment, with little rehabilitation. It is therefore not surprising that this experienced group of rheumatology OTs lacked confidence in VR.

On completion of the training programme, OTs’ knowledge and confidence improved significantly. The programme incorporated factual knowledge, workshops, discussion sessions, telephone workshops and tutorials – all designed to build on their existing expertise and transferable skills. Subsequently, they felt confident to carry out work assessments and identify work solutions and strategies.

The programme is three days long and includes self-study and a VR resource manual which includes easily accessible internet resources. Training materials have been developed which could be used in the future. The programme does not use any specialist equipment (only tools and equipment used in the workplace in practical sessions) or require specialist facilities, and it is easy to administer, rendering the programme low cost to deliver in any hospital or community setting. Although this training programme was designed specifically for OTs treating people with inflammatory arthritis, it could be modified to focus on job retention VR in general. Although a patient representative was included in the development of the programme, additional input from patient partners could be included to further develop the programme to identify specific strategies or equipment which they have found beneficial. They could also identify aspects of the programme which were likely to be less helpful to service users. Further development of the programme could include an additional day to focus on more practical skills which the OTs found most beneficial. This could potentially improve OTs’ VR knowledge and confidence in many clinical areas and have a positive impact on service users staying in work.

The limitations of the study are that the training programme was carried out with a small sample of specialist rheumatology OTs. The findings may not be representative of all OTs’ perceptions of their VR knowledge and confidence. A practical difficulty during training programme delivery was finding convenient dates for busy therapists (many of whom worked part time and had other work and family commitments) to attend, and some were unable to attend the follow-up day. Finally, although the OTs found the self-study activities to be beneficial, finding time to complete these during work time was difficult. They felt obligated to complete these in their own time and considered this a potential barrier to disseminating the training in its current form in practice.

## Conclusion

The main driver for this project was to train a specialist group of rheumatology OTs to deliver a VR intervention for people with inflammatory arthritis as part of an RCT. A training programme was developed primarily based on Allaire's VR intervention developed in the USA (2003), based on an understanding that the OTs’ current VR knowledge and skills are tailored to the UK; therefore, it focused on conducting a work assessment, practical training in VR strategies, and relevant UK employment and health and safety at work legislation. The programme led to significant improvements in the OTs’ VR knowledge and confidence. However, the results should be viewed with caution as the sample size was small. Further research, in a larger sample of rheumatology OTs, is needed to determine the effectiveness of the training programme. The programme could be modified to be relevant for other areas of clinical practice; this would require further development work.
